# COVID-19—does social distancing include species distancing?

**DOI:** 10.1007/s10460-020-10066-0

**Published:** 2020-06-08

**Authors:** Undine Giseke

**Affiliations:** Technische Universität Berlin, Berlin, Germany

*We* are disoriented. The most current reason for this is the COVID-19 pandemic, but our disorientation actually started long before. Let’s take the expression *We* as simply meaning “humankind”, and COVID-19 for the interaction between viruses and mammals, including humans. Right now *We* are being strongly influenced by global phenomena: The disorientation *We*'ve already experienced due to global warming, virtual transformation and the expansion of the technosphere has been increased by the addition of another form, i.e. the immediate disorientation caused by a virus.

*We* are stuck right in the middle, across borders—and responsible. When it comes to climate change *We* have always been (and still are) caught up in a discussion about whether *We* are already a part of it, but the coronavirus leaves no doubt about this: not in the way it acts or how enters our bodies, nor in the way it spreads globally. The virus ignores and transcends organic, continental and political boundaries. At the same time it closes borders; images of entire fleets of aircraft on the ground and travel bans are examples of this. These are indeed paradoxical phenomena, which exacerbate our disorientation and force us to immediately deal with an unknown. From a systemic perspective, it could be said that the virus unintentionally uses us as a system chain, with the result being that *We* have broken many other system chains that were considered untouchable up to now.

The dissolving of boundaries and the questioning of existing categories and systematics has preoccupied us for some time now. With the beginning of the new century and the concept of the Anthropocene, these issues have gained enormous momentum, both in terms of real life and scientific theory. And now, within a matter of weeks, the virus has become a marker, accelerator and spokesperson for the Anthropocene discourse.

What does the Anthropocene imply? It expresses the idea, in short, that the changes in the Earth system caused by humans are comparable to geological forces. Humans themselves—the global *We*—are believed to have become a geological factor. The concept of the Anthropocene (Crutzen 2002) has been taken up by many disciplines since the 2000’s and now encompasses different strands of discourse (Dürbeck 2018). It is undisputed that this new geological period is characterised by an extreme increase in human-induced, material exchange processes everywhere across the planet. It seems, however, that these calculations were made without considering non-human actors such as viruses.

With regards to climate change, *We* can now directly and physically experience the Anthropocene changes of a natural system that was long considered stable. The corona virus has forced us to quickly broaden our perspective. *We* are becoming conscious of the fact that the spread of the human species and its way of living is not only of a geological magnitude. Due to the spread of human-influenced habitats, the interaction between humans and animals is also being intensified, and thus, according to virologists, viruses can more easily change from animal hosts to humans. Just under half of the viral species found in mammals have so far been detected in humans, and most of them are a result of zoonosis.

The causes that have led to these intensified human-animal interactions are manifold, and they go hand in hand with our globalised, neoliberal way of life and economy. Population growth and migration, as well as the restriction of habitats for wild animals through an expansion of human activities are cited by scientist as reasons, as are the intensification of agriculture, changes in our food system, a mobility pattern that has lead to more intercontinental travel, a public health system that is desolate in many areas, and global warming.

So how do *We* proceed?

One consequence for the disciplines that design space must be on a higher level and fundamentally question the relationship between the co-existence of humans and other species and space (Renn 2017). While in the Anthropocene discourse a shift in the centrality of humans to the deep evolutionary processes of the earth's history, i.e. deep time, and the depth of the geological layers of the earth, i.e. geomorphology or deep ground (Wieck and Giseke 2018), has already begun, COVID-19 has forced us to significantly broaden our focus.

Technology and animals should be our family today, demands the feminist, biologist and utopian thinker (Haraway 2016) in a very radical, inspiring and hopeful way. In order that such future-oriented thinking is not led ad absurdum by COVID-19, *We* should regard the present situation as an indispensable challenge to radically broaden our frame of thought and to orient and apply our thinking and acting to the creation of connections. Where do *We* stand? Isn’t it absurd that the amount of chicken bones produced worldwide—which is breathtakingly high—was actually discussed as a marker for the scientific classification of the Anthropocene (Bennett et al. 2018). What do our global food systems look like? Currently, nearly 22 billion chickens coexist with us on this planet at any given time. The number of individual chicken slaughtered annually is almost three times as high, however. How will *We* co-exist with non-human species in the future? Are dairy cows merely a type of technology for us (Gan et al. 2016)? Scientists assume that flying foxes, which are possible carriers of coronaviruses, have played a central role in the settlement of so-called evolutionary hotspots, such as the volcanic islands in the Indian Ocean. Ambivalences of a multi-species entanglement.

At the current stage of being directly affected by the virus, *We* face other urgent issues as well: How do *We* protect ourselves, how do *We* slow down the spread of the virus, and how do *We* show solidarity in the fight against this virus? If *We* want to achieve co-existence, *We* must take a different long-term view of our vulnerability. Is there a *We* at all?

COVID-19 also ultimately reminds us of the manifold unsolved challenges of the porosity and closure of borders between different species, humans, animals and viruses, and of the organisation of space associated with them. It makes us aware of the daily mutual pervasiveness and linking of systems and system chains, intended or not. In questions of nutrition, everyday each of us is, in dependence on the global food system, a contributor and decision maker regarding our direct links to non-human species. This virus makes it clear to us that the times of uninhibited consumption are finally over, and that more space, both mentally and physically, must be given to questions of co-existence.
